# Harnessing the Qualities and Principles of Adult Education for Health Literacy Learning

**DOI:** 10.3928/24748307-20240613-03

**Published:** 2024-07

**Authors:** Danielle Marie Muscat

My doctoral degree involved the development and evaluation of a health literacy program for adult learners in Australia (McCaffery et al., 2019; Muscat et al., 2019; Muscat et al., 2020). It is unsurprising, then, that one of the first health literacy researchers I came to know of was Dr. Rima Rudd. It was through her work that I began reading about adult education institutions as a potential vehicle to build health literacy of those most in need and exploring the value of health-education partnerships (Rudd, 2002, 2004). Through this early reading, I was quickly linked through Dr. Rudd to the philosophies of Freire and others where I delved into problem-posing approaches to education and came to learn that adult education is rooted in a historical context concerned with capacity building, empowerment, and social change (McCaffery et al., 2006; Rudd & Comings, 1994). According to Critical Pedagogy theory, the role of education is to raise critical consciousness and develop strategies to overcome obstacles, including those to good health (Freire, 1974; Wallerstein & Bernstein, 1988). Quickly, my understanding of literacy and health literacy expanded well beyond a set of functional skills to a means to enable people to overcome disadvantage, control their lives, and become autonomous, participating citizens (Fajardo, 2015). Adult education institutions were positioned as a unique space to enable this.

Since I obtained my doctoral degree, my work in health literacy has moved out of the adult education sphere and more toward health systems, where I have adapted the original adult education program for different community and clinical populations, including adults living with chronic kidney disease (Muscat, Lambert, et al., 2021) and new parents (Muscat, Ayre, et al., 2021). This process has made me appreciate—possibly more than ever—Dr. Rudd's reflections about the unique space that adult education institutions offer for health literacy learning. Delivering programs within health care spaces that are necessarily time-limited and with still few opportunities for people to connect as a group of learners, I have come to appreciate the luxury of having had 18 weeks of ongoing, face-to-face engagement with learners, facilitated by a trained adult education teacher with expertise in literacy development.

So, my first call to action is more of an echo of one that Dr. Rudd made many years ago; to continue to harness adult education institutions as a space to develop skills among members of the general public to improve health literacy (Office of the Surgeon General; Office of Disease Prevention and Health Promotion, 2006). In Australia, adult education institutions have national reach and provide learning opportunities for adults with social disadvantages with lower literacy and numeracy, including a high proportion of women, those who are unemployed, people from culturally and linguistically diverse backgrounds and those with low socioeconomic status and from regional and remote areas (TAFE NSW, 2015). However, vocational training reforms across all Australian states have resulted in a decrease in funding and an increased emphasis on vocational skills (Needham, 2015), with what some educationalists have labeled a “narrow and behaviourist” focus on workplace tasks and roles (Wheelahan, 2015b). We must continue to advocate for a broader, more holistic notion of adult education which aims to facilitate learners' civic and personal development (Wheelahan, 2015a) and for the removal of the “institutional silos” between education and health sectors to support the integration of health-focused programs.

But alongside this call, can we also harness the qualities and underpinning foundations of adult education across settings? Dr. Rudd's work reminds us of how this might be achieved. As a starting point, across all settings, health literacy programs can emphasize health-related tasks and related literacy skills. These include, for example, skills related to health system vocabulary, dialogue, and discussion with health providers (Rudd, 2002), and increasingly in critical appraisal of health information. Our own work has shown that embedding skills within health topics of interest may be an opportune way to achieve this in health systems where health literacy programs “compete” in an already crowded space of health communication and information transfer (Muscat, Ayre, et al., 2021; Zwi et al., 2022).

Second, educational philosophies can help to inform the design of health literacy programs, including those delivered within the health system. Problem-posing education, for example, seeks to support adult learners to become reflective, critical thinkers (Rudd & Comings, 1994). It stands in opposition to more passive educational models where the teacher makes all decisions and remains the subject of the learning process (Rudd & Comings, 1994). Building on problem-posing education models, health literacy programs can—as a first step—seek to breakdown the dichotomy between student and teacher and establish a situation of equality, dialogue, and mutual communication (Rudd & Comings, 1994). Programs can then be designed to offer problem-posing and problem-solving opportunities and to include the active participation of learners in the production of learning materials (Rudd & Comings, 1994).

Finally, much literature on adult and community education focuses on the creation of open spaces to support the development of cohesive communities in which people feel comfortable and involved. Such “co-learning” usually involves creating a learning environment where learners can interact and exchange health knowledge and experiences with family, community members or peers alongside the facilitator to learn from each other (de Wit et al., 2017). Positioning “co-learning” as integral to health literacy programs both as a method and outcome is an important goal across settings. Facilitating this is increasingly achievable in online settings.

## Conclusion

I would be remiss not to acknowledge Dr. Rudd's later work, where she pioneered organizational and system-level responses to remove literacy-related barriers to health, shifting—as she has described—“the spotlight from the literacy skills of the public to the activities of health systems and health care professionals” (Rudd, 2010, p. 2283). Indeed, this has been instrumental in shaping the field and has offered insights for efficacious action, which have led to tangible health system improvements (Rudd, 2013). But, for me, her work in adult education will always hold an important place. I value this opportunity to remind people that it should not be ignored, alongside our organizational-level and systems approaches.
